# Synthesis and biological profile of substituted benzimidazoles

**DOI:** 10.1186/s13065-018-0498-y

**Published:** 2018-12-01

**Authors:** Neelam Vashist, Surinder Singh Sambi, Balasubramanian Narasimhan, Sanjiv Kumar, Siong Meng Lim, Syed Adnan Ali Shah, Kalavathy Ramasamy, Vasudevan Mani

**Affiliations:** 1 SGT College of Pharmacy, Shree Guru Gobind Singh Tricentenary (SGT) University, Gurugram, 122505 India; 2 University School of Chemical Technology, Guru Gobind Singh Indraprastha University, Sector-16C, Dwarka, New Delhi 110078 India; 3 Faculty of Pharmaceutical Sciences, Maharshi Dayanand University, Rohtak, 124001 India; 4 Faculty of Pharmacy, Universiti Teknologi MARA (UiTM), 42300 Bandar Puncak Alam, Selangor Darul Ehsan Malaysia; 5 Collaborative Drug Discovery Research (CDDR) Group, Pharmaceutical Life Sciences Community of Research, Universiti Teknologi MARA (UiTM), 40450 Shah Alam, Selangor Darul Ehsan Malaysia; 6 Atta-ur-Rahman Institute for Natural Products Discovery (AuRIns), Universiti Teknologi MARA, Puncak Alam Campus, 42300 Bandar Puncak Alam, Selangor Darul Ehsan Malaysia; 7 Department of Pharmacology and Toxicology, College of Pharmacy, Qassim University, Buraidah, 51452 Kingdom of Saudi Arabia

**Keywords:** Benzimidazoles, Synthesis, Antimicrobial activity, Anticancer activity

## Abstract

**Background:**

A series of benzimidazole derivatives was developed and its chemical scaffolds were authenticated by NMR, IR, elemental analyses and physicochemical properties. The synthesized compounds were screened for their antimicrobial and antiproliferative activities.

**Results and discussion:**

The synthesized benzimidazole compounds were evaluated for their antimicrobial activity using the tube dilution method and were found to exhibit good antimicrobial potential against selected Gram negative and positive bacterial and fungal species. The compounds were also assessed for their anticancer activity exhibited using the SRB assay and were found to elicit antiproliferative activity against MCF7 breast cancer cell line, which was comparable to the standard drug.

**Conclusion:**

Antimicrobial screening results indicated that compounds **1**, **2** and **19** to be promising antimicrobial agents against selected microbial species and comparable to standard drugs which included norfloxacin and fluconazole. The anticancer screening results revealed that compounds, **12**, **21**, **22** and **29** to show the highest activity against MCF7 and their IC_50_ values were more potent than 5-fluorouracil.
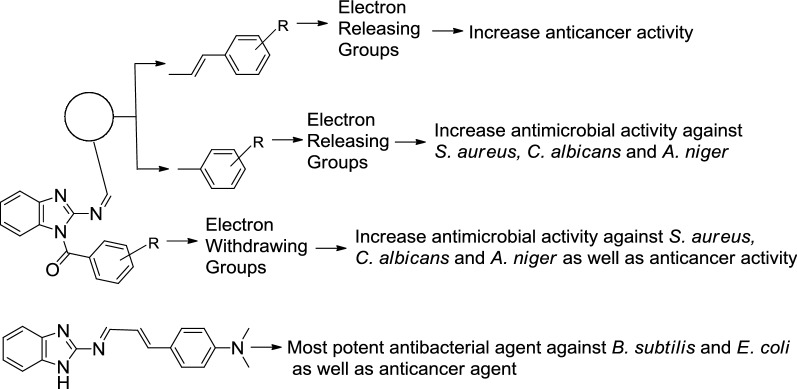

## Background

The emergence of antibiotic-resistant microorganisms such as fluoroquinolone-resistant *Escherichia coli, Streptococcus pneumonia,* carbapenem-resistant *Klebsiella pneumonia*, vancomycin-resistant *enterococci* and methicillin-resistant *Staphylococcus aureus* is becoming a serious health issue worldwide. There is a critical need to develop new chemotherapeutic agents with different mechanism of action [1].

Cancer is a deadly disease prevalent in both the developing as well as the developed countries. In spite of significant improvements in recognition and treatment of cancer, the incidence of certain types of malignancy is still on the rise. Current treatments such as cytotoxic chemotherapy and radiotherapy yielded only transient therapeutic aids that are accompanied by severe adverse effects. This is due to their toxic effects against normal growing cells. Concerted effort is, therefore, required to eliminate or at least reduce these incidences significantly [2].

Recent findings suggest that substituted benzimidazole derivatives possess potential chemotherapeutic activity with reduced toxic effects. Antibacterial activity of substituted benzimidazole derivatives can be explained by their competition with purines, an integral part of bacterial strain, resulting in inhibition of bacterial nucleic acids and proteins synthesis [3]. Compounds containing benzimidazole moiety such as thiabendazole, parbendazole, mebendazole, albendazole, cambendazole and flubendazole had also been reported for their antihelminthic activity. Similarly, the proton pump inhibitors, omeprazole, lansoprazole, rabeprazole, pantoprazole, had been reported for their use in the management of acid related disorders. In fact, benzimidazole derivatives had found their applications as antioxidant [4], antimicrobial [5], antihelmintic [6], anticancer [7], antiviral [8], antiallergic [9], antiarthritic [10] and anti-mycobacterial agents [11].

In light of above, the present study was undertaken to synthesise and evaluate the antimicrobial and anticancer potentials of substituted benzimidazole derivatives.

## Results and discussion

### Chemistry

**Scheme 1:**
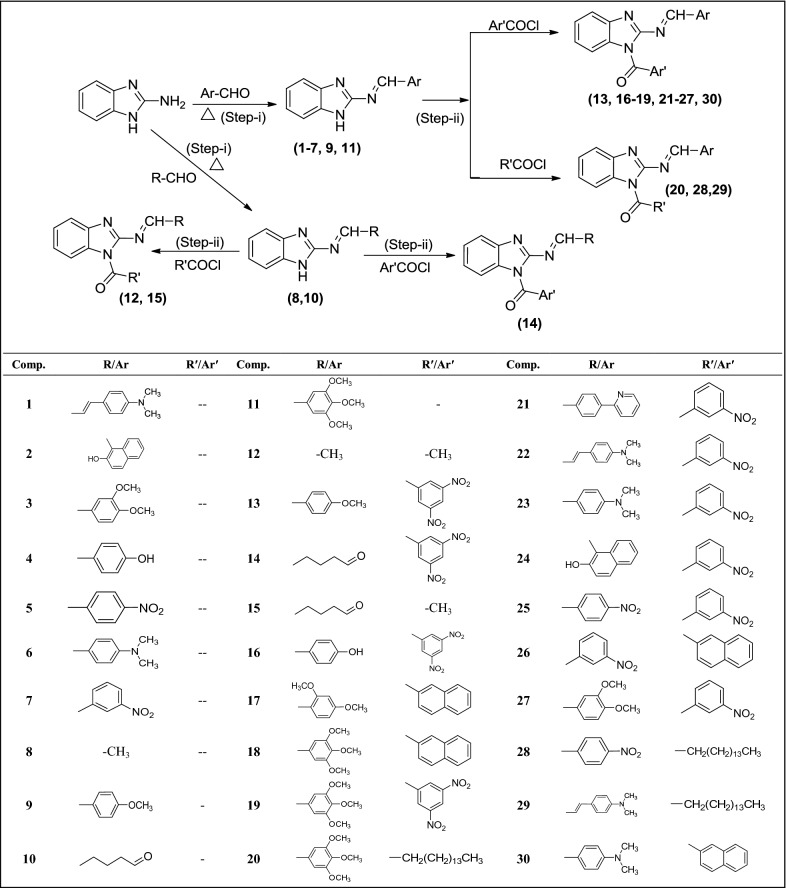
Synthesis of benzimidazole derivatives (**1**–**30**). Reaction condition: Step i: 2-Aminobenzimidazole, substituted aldehyde, ethanol, glacial acetic acid, reflux for 4–5 h (RT), Step ii: Schiff’s base, different acylchlorides, dimethylformamide, triethylamine, stir for 24 h (RT)

Target compounds (**1**–**30**) were synthesized by following procedure outlined in Scheme 1. The physicochemical data of the target compounds are presented in Table 1. The synthesized compounds were evaluated on the basis of spectral analysis: IR, NMR and Mass and elemental analyses which were in full agreement with their proposed molecular structures. The formation of Schiff bases is confirmed by the presence of N=CH str., at around 1560 cm^−1^ in the IR spectra of synthesized compounds (**1**–**11**). Asym str., at around 1550 cm^−1^ indicated the presence of aromatic nitro group in **5**, **7**, **13**, **14**, **16**, **21-28** compounds. The presence of C–O–C str., of aralkyl showed methoxy group in **3, 9, 11, 13, 17**–**20**, **27** compounds. The C–H str., at 1727 cm^−1^ confirmed the aliphatic aldehyde group in **10, 14** and **15** compounds. Furthermore, the appearance of C=O str., at 1660 cm^−1^ and the absence of NH str., of imidazole at 3400 cm^−1^ confirmed the synthesis of methanone derivatives (**12**–**30**). The multiplet corresponds to 6.697–7.823 δ ppm confirmed the presence of aromatic protons of aryl nucleus and benzimidazole. The appearance of singlet at around 9.580 δ ppm confirmed the Schiff bases (N=CH–). The singlet peak at 3.426 δ ppm indicated the presence of dimethyl group in compounds, **1, 6, 22, 29** and **30**. The doublet peak observed at 1.273–1.276 δ ppm which confirmed the presence of aliphatic methyl group in the synthesized compounds, **8**, **12** and **15**. The multiplet showed at 1.243–2.496 δ ppm confirmed the presence of CH_2_ chain of palmitoyl group in the structure of compounds (**20**, **28** and **29**). Further confirmation was made on the basis of ^13^C-NMR and MS spectral analyses. The results of C, H, N analysis are within limits of ± 0.3%.

**Table 1:** Physicochemical characteristic of the synthesized compounds

Comp.	Molecular structures with stereochemistry	M. formula and CHN analyses	M. wt.	R*f* value	% Yield	M. Pt. (°C)
**1**	*(E)*-*N*-*((E)*-*3*-*(4*-*(Dimethylamino)phenyl) allylidene)*-*1H*-*benzo[d]imidazol*-*2*-*amine*	C_18_H_18_N_4_: Anal calcd: C, 74.46; H, 6.25; N, 19.30; Found: C, 74.43; H, 6.27; N, 19.33	290.40	0.76^a^	76	228–230
**2**	*(E)*-*1*-*(((1H*-*Benzo[d]imidazol*-*2*-*yl)imino)methyl)naphthalen*-*2*-*ol*	C_18_H_13_N_3_O: Anal calcd: C, 75.25; H, 4.56; N, 14.63; Found: C, 75.27; H, 4.59; N, 14.60	287.34	0.79^a^	74	255–257
**3**	*(E)*-*N*-*(3,4*-*Dimethoxybenzylidene)*-*1H*-*benzo[d]imidazol*-*2*-*amine*	C_16_H_15_N_3_O_2_: Anal calcd: C, 68.31; H, 5.37; N, 14.94; Found: C, 68.34; H, 5.35; N, 14.97	281.34	0.77^a^	78	225–227
**4**	*(E)*-*4*-*(((1H*-*Benzo[d]imidazol*-*2*-*yl)imino)methyl)phenol*	C_14_H_11_N_3_O: Anal calcd: C, 70.87; H, 4.67; Cl, 17.71; Found: C, 70.88; H, 4.65; Cl, 17.73	237.28	0.75^a^	67	220–222
**5**	*(E)*-*N*-*(4*-*Nitrobenzylidene)*-*1H*-*benzo[d]imidazol*-*2*-*amine*	C_14_H_10_N_4_O_2_: Anal calcd: C, 63.15; H, 3.79; N, 21.04; Found: C, 63.13; H, 3.77; N, 21.07	266.28	0.72^a^	72	236–238
**6**	(*E)*-*N*-*(4*-*(Dimethylamino)benzylidene)*-*1H*-*benzo[d]imidazol*-*2*-*amine*	C_16_H_16_N_4_: Anal calcd: C, 72.70; H, 6.10; N, 21.20; Found: C, 72.73; H, 6.12; N, 21.22	264.36	0.79^a^	82	238–240
**7**	*(E)*-*N*-*(3*-*Nitrobenzylidene)*-*1H*-*benzo[d]imidazol*-*2*-*amine*	C_14_H_10_N_4_O_2_: Anal calcd: C, 63.15; H, 3.79; N, 21.04; Found: C, 63.18; H, 3.77; N, 21.05	266.28	0.76^a^	76	190–192
**8**	*(E)*-*N*-*Ethylidene*-*1H*-*benzo* *[d]imidazol*-*2*-*amine*	C_9_H_9_N_3_: Anal calcd: C, 67.90; H, 5.70; N, 26.40; Found: C, 67.88; H, 5.72; N, 26.42	159.21	0.72^a^	80	172–175
**9**	*(E)*-*N*-*(4*-*Methoxybenzylidene)*-*1H*-*benzo[d]imidazol*-*2*-*amine*	C_15_H_13_N_3_O: Anal calcd: C, 71.70; H, 5.21; N, 16.72; Found: C, 71.73; H, 5.22; N, 16.74	251.31	0.71^a^	75	198–200
**10**	*(E)*-*5*-*((1H*-*Benzo[d]imidazol*-*2*-*yl)imino)pentanal*	C_12_H_13_N_3_O: Anal calcd: C, 66.96; H, 6.09; N, 19.52; Found: C, 66.94; H, 6.11; N, 19.55	215.28	0.75^a^	78	265–267
**11**	*(E)*-*N*-*(3,4,5*-*Trimethoxybenzylidene)*-*1H*-*benzo[d]imidazol*-*2*-*amine*	C_17_H_17_N_3_O_3_: Anal calcd: C, 65.58; H, 5.50; N, 13.50; Found: C, 65.61; H, 5.53; N, 13.52	311.37	0.72^a^	84	242–245
**12**	*(E)*-*1*-*(2*-*(Ethylideneamino)*-*1H*-*benzo[d]imidazol*-*1*-*yl)ethanone*	C_11_H_11_N_3_O: Anal calcd: C, 65.66; H, 5.51; N, 20.88; Found: C, 65.65; H, 5.54; N, 20.86	201.22	0.63^b^	74	262–265
**13**	*(E)*-*(3,5*-*Dinitrophenyl)(2*-*((4*-*methoxy*-*benzylidene)amino)*-*1H*-*benzo[d]imidazol*-*1*-*yl)methanone*	C_22_H_15_N_5_O_6_: Anal calcd: C, 59.33; H, 3.39; N, 15.72; Found: C, 59.35; H, 3.42; N, 15.75	445.38	0.58^b^	68	243–245
**14**	(*E)*-*5*-*((1*-*(3,5*-*Dinitrobenzoyl)*-*1H*-*benzo[d]imidazol*-*2*-*yl)imino)pentanal*	C_14_H_11_N_3_O_2_: Anal calcd: C, 55.75; H, 3.69; N, 17.11; Found: C, 55.78; H, 3.71; N, 17.14	253.26	0.66^b^	65	162–164
**15**	*(E)*-*5*-*((1*-*Acetyl*-*1H*-*benzo[d]imidazol*-*2*-*yl)imino)pentanal*	C_14_H_15_N_3_O_2_: Anal calcd: C, 65.35; H, 5.88; N, 16.33; Found: C, 65.37; H, 5.90; N, 16.36	257.29	0.62^b^	72	226–228
**16**	*(E)*-*(3,5*-*Dinitrophenyl)(2*-*((4*-*hydroxybenzylidene)amino)*-*1H*-*benzo[d]imidazol*-*1*-*yl)methanone*	C_21_H_13_N_5_O_6_: Anal calcd: C, 58.47; H, 3.04; N, 16.24; Found: C, 58.49; H, 3.05; N, 16.25	431.36	0.64^b^	70	175–177
**17**	(*E)*-*(2*-*((2,4*-*Dimethoxybenzylidene)amino)* -*1H*-*benzo[d]imidazol*-*1*-*yl)(naphthalen*-*2*-*yl)methanone*	C_27_H_21_N_3_O_3_: Anal calcd: C, 74.47; H, 4.86; N, 9.65; Found: C, 74.49; H, 4.88; N, 9.68	435.47	0.54^b^	67	120–122
**18**	*(E)*-*Naphthalen*-*2*-*yl(2*-*((3,4,5*-*trimethoxybenzylidene)amino)*-*1H*-*benzo[d]imidazol*-*1*-*yl)methanone*	C_28_H_23_N_3_O_4_: Anal calcd: C, 72.24; H, 4.98; N, 9.03; Found: C, 72.27; H, 4.95; N, 9.05	465.5	0.65^b^	75	210–212
**19**	*(E)*-*(3,5*-*Dinitrophenyl)(2*-*((3,4,5*-*trimethoxybenzylidene)amino)*-*1H*-*benzo[d]imidazol*-*1*-*yl)methanone*	C_24_H_19_N_5_O_8_: Anal calcd: C, 57.03; H, 3.79; N, 13.86; Found: C, 57.07; H, 3.76; N, 13.88	505.44	0.66^b^	66	141–143
**20**	*(E)*-*1*-*(2*-*((3,4,5*-*trimethoxybenzylidene)amino)*-*1H*-*benzo[d]imidazol*-*1*-*yl)hexadecan*-*1*-*one*	C_33_H_47_N_3_O_4_: Anal calcd: C, 72.10; H, 8.62; N, 7.64; Found: C, 72.11; H, 8.65; N, 7.67	549.74	0.62^b^	78	136–138
**21**	*(E)*-*(3*-*Nitrophenyl)(2*-*((4*-*(pyridin*-*2*-*yl)benzylidene)amino)*-*1H*-*benzo[d]imidazol*-*1*-*yl)methanone*	C_26_H_17_N_5_O_3_: Anal calcd: C, 69.79; H, 3.83; N, 15.65; Found: C, 69.77; H, 3.86; N, 15.68	447.44	0.57^b^	67	142–144
**22**	*(2*-*((E)*-*((E)*-*3*-*(4*-*(Dimethylamino)phenyl) allylidene)amino)*-*1H*-*benzo[d]imidazol*-*1*-*yl)(3*-*nitrophenyl)methanone*	C_25_H_21_N_5_O_3_: Anal calcd: C, 68.33; H, 4.82; N, 15.94; Found: C, 68.37; H, 4.80; N, 15.97	439.47	0.59^b^	82	126–128
**23**	(*E)*-*(2*-*((4*-*(Dimethylamino)benzylidene) amino)*-*1H*-*benzo[d]imidazol*-*1*-*yl)(3*-*nitrophenyl)methanone*	C_23_H_19_N_5_O_3_: Anal calcd: C, 66.82; H, 4.63; N, 16.94; Found: C, 66.83; H, 4.66; N, 16.97	413.43	0.63^b^	76	131–133
**24**	*(E)*-*(2*-*(((2*-*hydroxynaphthalen*-*1*-*yl)*-*methylene)amino)*-*1H*-*benzo[d]imidazol*-*1*-*yl)(3*-*nitrophenyl)methanone*	C_25_H_16_N_4_O_4_: Anal calcd: C, 68.80; H, 3.70; N, 12.84; Found: C, 68.83; H, 3.72; N, 12.87	436.42	0.64^b^	65	134–136
**25**	*(E)*-*(2*-*((4*-*Nitrobenzylidene)amino)*-*1H*-*benzo[d]imidazol*-*1*-*yl)(3*-*nitrophenyl) methanone*	C_21_H_13_N_5_O_5_: Anal calcd: C, 60.72; H, 3.15; N, 16.86; Found: C, 60.75; H, 3.17; N, 16.89	415.36	0.66^b^	74	119–121
**26**	*(E)*-*Naphthalen*-*2*-*yl(2*-*((4*-*nitrobenzylidene)amino)*-*1H*-*benzo[d]imidazol*-*1*-*yl)methanone*	C_25_H_16_N_4_O_3_: Anal calcd: C, 71.42; H, 3.84; N, 13.33; Found: C, 71.43; H, 3.87; N, 13.37	420.42	0.52^b^	62	176–178
**27**	(*E)*-*(2*-*((3,4*-*Dimethoxybenzylidene)amino)* -*1H*-*benzo[d]imidazol*-*1*-*yl)(3*-*nitro phenyl)methanone*	C_23_H_18_N_4_O_5_: Anal calcd: C, 64.18; H, 4.22; N, 13.02; Found: C, 64.21; H, 4.25; N, 13.04	430.41	0.63^b^	65	235–237
**28**	*(E)*-*1*-*(2*-*((4*-*Nitrobenzylidene)amino)*-*1H*-*benzo[d]imidazol*-*1*-*yl)hexadecan*-*1*-*one*	C_30_H_40_N_4_O_3_: Anal calcd: C, 71.40; H, 7.99; N, 11.10; Found: C, 71.39; H, 7.97; N, 11.12	504.66	0.59^b^	66	126–128
**29**	*(E)*-*1*-*(2*-*((4*-*(Dimethylamino)benzylidene) amino)*-*1H*-*benzo[d]imidazol*-*1*-*yl)hexadecan*-*1*-*one*	C_34_H_48_N_4_O: Anal calcd: C, 77.23; H, 9.15; N, 10.60; Found: C, 77.21; H, 9.16; N, 10.63	528.77	0.64^b^	68	131–133
**30**	*(E)*-*(2*-*((4*-*(Dimethylamino)benzylidene) amino)*-*1H*-*benzo[d]imidazol*-*1*-*yl)(naphthalen*-*2*-*yl)methanone*	C_27_H_22_N_4_O: Anal calcd: C, 77.49; H, 5.30; N, 13.39; Found: C, 77.51; H, 5.32; N, 13.42	418.49	0.62^b^	76	192–195

TLC mobile phase: ^a^ Ethyl acetate: Methanol (7:3); ^b^ Chloroform: Methanol (8:2)

### Anticancer activity

The synthesized benzimidazole derivatives were screened for their anticancer activity against MCF7 (ATCC HTB-22), an oestrogen receptor positive human breast adeno-carcinoma cell line. Anticancer screening results (Table 2) indicated that compound **22** (IC_50_ = 0.9 µM) was found to be the most potent when compared to the standard drug, 5-fluorouracil (IC_50_ = 35.4 µM). Other compounds which included **12**, **21** and **29** also exhibited more potent antiproliferative results (IC_50_ = 7.0, 5.4 and 5.5 µM, respectively) when compared to the standard drug. These compounds may be used as drug leads for discovery of new anticancer agents.

**Table 2:** Anticancer screening results of synthesized compounds

Comp.	MCF-7 cell line	Comp.	MCF-7 cell line
*Anticancer screening (IC*_*50*_ = µM*)*			
**1**	31.0	**16**	231.8
**2**	41.8	**17**	39.0
**3**	170.6	**18**	161.1
**4**	101.1	**19**	15.8
**5**	67.6	**20**	23.6
**6**	> 378.3	**21**	5.4
**7**	112.7	**22**	00.9
**8**	> 628.1	**23**	50.8
**9**	> 310.4	**24**	61.9
**10**	157.9	**25**	18.8
**11**	> 321.2	**26**	176.0
**12**	7.0	**27**	123.1
**13**	19.1	**28**	31.7
**14**	> 394.9	**29**	5.5
**15**	11.7	**30**	138.6
5-Fluorouracil	35.4	5-Fluorouracil	35.4

### Antimicrobial activity

Antimicrobial activity results (Table 3) indicated that the compounds possessed good antimicrobial activity against the tested bacterial and fungal strains. Compound **1** showed good antibacterial activity against *E. coli* (MIC_*ec*_ = 5.4 µM) and *B. subtilis* (MIC_*bs*_ = 10.7 µM), whereas compound **19** was found to be more potent against *S. aureus* (MIC_*sa*_= 12.4 µM). The reference drug, norfloxacin, yielded MIC of 4.7 µM against the tested microorganisms. The antifungal activity results indicated that compound **2** showed good activity against *C. albicans* (MIC_*ca*_ = 5.4 µM. Compound **19**, on the other hand, was the most potent antifungal agent against *A. niger* (MIC_*an*_ = 3.1 µM) in comparison to fluconazole (MIC = 5.0 µM), the reference drug. Thus, compound **19** may serve as a potential lead compound for the design of novel antifungal agents.

**Table 3:** Antimicrobial activity of synthesized compounds

Comp.	Antimicrobial screening (MIC = µM)				
	Bacterial species			Fungal species	
	*E. coli*	*B. subtilis*	*S. aureus*	*C. albicans*	*A. niger*
**1**	05.4	10.7	43	10.7	21.5
**2**	43.5	21.8	43.5	5.4	10.9
**3**	22.2	22.2	44.4	11.1	22.2
**4**	06.6	13.1	52.7	13.1	26.3
**5**	46.9	23.5	46.9	23.5	23.5
**6**	47.3	23.6	47.3	47.3	47.3
**7**	23.5	23.5	46.9	93.9	23.5
**8**	39.3	39.3	78.5	78.5	78.5
**9**	24.9	24.9	49.7	24.9	49.7
**10**	14.5	29	58.1	29	58.1
**11**	10.0	20.1	40.1	20.1	20.1
**12**	248.5	248.5	15.5	62.1	31.1
**13**	56.1	28.1	14	14	7
**14**	197.4	197.4	24.7	98.7	24.7
**15**	194.3	194.3	24.3	48.6	24.3
**16**	58.0	29	14.5	14.5	7.2
**17**	28.7	28.7	14.4	28.7	7.2
**18**	26.9	13.4	13.4	26.9	6.7
**19**	49.5	12.4	12.4	6.2	3.1
**20**	45.5	11.4	22.7	22.7	11.4
**21**	27.9	14	14	55.9	7
**22**	56.9	28.4	14.2	28.4	7.1
**23**	60.5	30.2	15.1	30.2	7.5
**24**	28.6	28.6	14.3	28.6	7.1
**25**	60.2	30.1	15	30.1	7.5
**26**	29.7	29.7	14.9	59.5	7.4
**27**	58.1	29	14.5	29	7.2
**28**	49.5	24.8	24.8	24.8	12.4
**29**	47.3	11.8	23.6	23.6	11.8
**30**	29.9	29.9	14.9	59.7	7.5
**DMSO**	NA	NA	NA	NA	NA
**Std. drugs**	4.7^a^	4.7^a^	4.7^a^	5.1^b^	5.1^b^

*NA* no activity, *DMSO* dimethyl sulphoxide

Std. drugs: ^a^ Norfloxacin, ^b^ Fluconazole

## Structure activity relationship

**Fig. 1:**
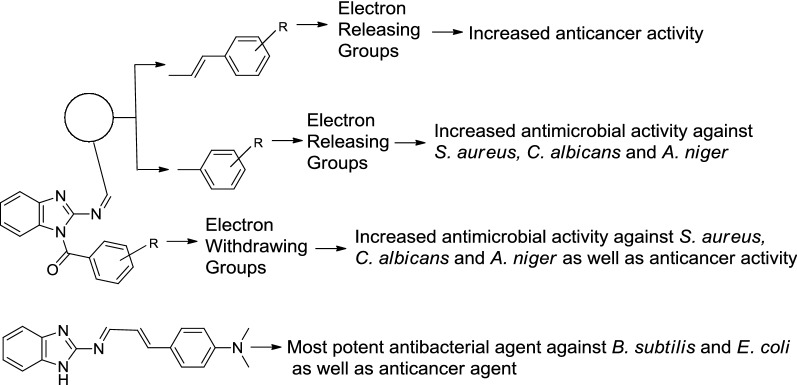
Structural requirements for antimicrobial and anticancer activity of synthesized benzimidazole derivatives

The following structure activity relationship may be drawn from the antimicrobial and anticancer activities of the benzimidazole derivatives (Fig. 1):

- It has been noticed that the antibacterial activity of Schiff bases against *E. coli* enhanced due to the presence of vinyl group between benzimidazole amine and *N*-benzylidene moiety and the substitution of electron releasing group at phenyl nucleus as in the compound **1** and the same moiety improved anticancer activity of methanone derivatives as in compound **22**.
- The electron donating group placed at phenyl ring attached to *N*-alylidene/arylidene moiety along with presence of electron withdrawing group on phenyl ring attached to methanone moiety improved antibacterial and antifungal activity of synthesized benzimidazole derivatives against bacterial and fungal strains as in compound **19**.

## Experimental

### Materials and methods

All the laboratory reagents were procured from Sigma Aldrich and were used without any purification. Melting points were determined on Sonar melting point apparatus in an open capillary tube and are uncorrected. Purity of the compound was ascertained by commercialized (E-Merck Kieselgel 60 F254) TLC plates. The Infrared spectrum was recorded in KBr discs on a Shimadzu-FTIR 8400S spectrometer (ν_max_ in cm^−1^). Proton and

^13^C NMR spectra of the synthesized compounds were recorded on Bruker Advance-II 400 NMR spectrometer with DMSO as a solvent and the chemical shift data were expressed as delta values related to tetramethylsilane. Mass spectra were recorded using Waters, Q-TOF micromass spectrometer.

#### Procedure for the title compounds (**1**–**11**)

2-Aminobenzimidazole (0.01 mol) was refluxed with different substituted aromatic aldehyde (0.01 mol) in ethanol (20 ml) for 4–5 h (RT) in presence of glacial acetic acid (few drops). Then the reaction mixture was allowed to cool at RT and the precipitated compound was filtered and dried [12].

#### Synthesis of 2-(alkyl/arylideneamino)-1H-benzo[d]imidazol-1-yl-alkyl/aryl-methanones (**12**–**30**)

Compound of *Schiff’s bases* (**1**–**11**) (0.005 mol) were stirred at RT with different acylchlorides (0.005 mol) in dimethylformamide for 24 h with the addition of small amount of triethylamine. The resulting reaction mixture was precipitated using ice cold water and the crude product was filtered through a vacuum pump, washed with cold water, dried and recrystallized using rectified spirit [13].

#### Spectral data of synthesized compounds

##### *(E)*-*N*-*((E)*-*3*-*(4*-*(Dimethylamino)phenyl)allylidene)*-*1H*-*benzo[d]imidazol*-*2*-*amine* (**1**)

IR (KBr cm^−1^): 1550 (N=CH str.), 3475 (N–H str.), 1431 (Ar., C=C str.), 1253 (C–N str.), 1300 (–N(CH_3_)_2_ str); ^1^H NMR (DMSO): 9.557–9.575 (d, 1H, N=CH), 6.646–6.714 (d, 1H, –CH=CH), 6.416–7.614 (m, 8H, ArH), 3.426 (s, 6H, (CH_3_)_2_); ^13^C NMR (DMSO): 40, 115, 119, 123, 127, 135, 138, 148, 159, 162; MS: *m/z* = 291.12 (M^+^ +1).

##### *(E)*-*1*-*(((1H*-*Benzo[d]imidazol*-*2*-*yl)imino)methyl)naphthalen*-*2*-*ol* (**2**)

IR (KBr cm^−1^): 3066 (N–H str., of imidazole), 3012 (C–H aromatic ring str.), 1442 (Ar., C=C str.), 1550 (N=CH str.), 1253 (C–N str.), 3518 (O–H str.); ^1^H NMR (DMSO): 10.295 (s, 1H, N=CH), 7.096–8.108 (m, 10H, ArH), 4.481 (s, 1H, OH), 10.809 (s,1H, NH of imidazole); ^13^C NMR (DMSO): 115, 118, 123, 127, 128, 129, 132, 135, 138, 159, 162; MS: *m/z* = 288.39 (M^+^ +1).

##### *(E)*-*N*-*(3,4*-*Dimethoxybenzylidene)*-*1H*-*benzo[d]imidazol*-*2*-*amine* (**3**)

IR (KBr cm^−1^): 3410 (N–H str.), 3058 (Ar., C–H str.), 1542 (C=C str.), 1610 (N=CH str.), 2827 (Ar., OCH_3_ str.); ^1^H NMR (DMSO): 9.487 (s, 1H, N=CH), 6.982–7.849 (m, 7H, ArH), 10.452 (s, 1H, NH of imidazole) 3.502 (s, 6H, (OCH_3_)_2_); ^13^C NMR (DMSO): 56, 115, 123, 127, 138, 152, 159, 162; MS: *m/z* = 282.14 (M^+^ +1).

##### *(E)*-*4*-*(((1H*-*Benzo[d]imidazol*-*2*-*yl)imino)methyl)phenol* (**4**)

IR (KBr cm^−1^): 3440 (N–H str.), 3063 (Ar., C–H str.), 1537 (C=C str.), 1613 (N=CH str.), 3452 (O–H str.); ^1^H NMR (DMSO): 9.582 (s, 1H, N=CH), 7.106–8.367 (m, 8H, ArH), 10.809 (s, 1H, NH of imidazole); ^13^C NMR (DMSO): 115, 117, 123, 126, 129, 131, 138, 159, 162; MS: *m/z* = 238.17 (M^+^ +1).

##### *(E)*-*N*-*(4*-*Nitrobenzylidene)*-*1H*-*benzo[d]imidazol*-*2*-*amine* (**5**)

IR (KBr cm^−1^): 3240 (N–H str., of imidazole ring), 2974 (C–H aromatic ring str.), 1465 (Ar., C=C str.), 1550 (N=CH str.), 1548 (Ar–C–NO_2,_ asym str.); ^1^H NMR (DMSO): 9.550 (s, 1H, N=CH), 7.103–8.105 (m, 4H, ArH), 8.116–8.376 (d, 4H, Ar-NO_2_), 12.73 (s, 1H, NH of imidazole); ^13^C NMR (DMSO): 115, 120, 123, 130, 138, 149, 159, 162; MS: *m/z* = 267.26 (M^+^ +1).

##### *(E)*-*N*-*(4*-*(Dimethylamino)benzylidene)*-*1H*-*benzo[d]imidazol*-*2*-*amine* (**6**)

IR (KBr cm^−1^): 1550 (N=CH str.), 3374 (N–H str.), 1462 (Ar., C=C str.), 1298 (C–N str. –N(CH_3_)_2_); ^1^H NMR (DMSO): 9.206 (s, 1H, N=CH), 6.697–7.823 (m, 8H, ArH), 3.043 (s, 6H, (CH_3_)_2_), 12.42 (s, 1H, NH of imidazole); ^13^C NMR (DMSO): 40, 115, 123, 138, 159, 162; MS: *m/z* = 265.35 (M^+^ +1).

##### *(E)*-*N*-*(3*-*Nitrobenzylidene)*-*1H*-*benzo[d]imidazol*-*2*-*amine* (**7**)

IR (KBr cm^−1^): 3428 (N–H str.), 3068 (Ar., C–H str.), 1531 (C=C str.), 1618 (C=N str.), 1547 (Ar-NO_2_ str.); ^1^H NMR (DMSO): 9.515 (s, 1H, N=CH), 7.213–8.378 (m, 8H, ArH), 10.23 (s, 1H, NH of imidazole); ^13^C NMR (DMSO): 115,123, 127, 135, 138, 150, 159, 162; MS: *m/z* = 267.28 (M^+^ +1).

##### *(E)*-*N*-*Ethylidene*-*1H*-*benzo[d]imidazol*-*2*-*amine* (**8**)

IR (KBr cm^−1^): 3267 (N–H str., of imidazole ring), 2924 (C–H aromatic ring str.), 1465 (Ar., C=C str.), 1550 (N=CH str.), 2877 (R-CH_3_, sym str.); ^1^H NMR (DMSO): 8.654 (s, 1H, N=CH), 6.897–7.143 (m, 4H, ArH), 1.243 (s, 3H, CH_3_); ^13^C NMR: 22, 111, 120, 154, 175. MS: *m/z* = 160.28 (M^+^ +1).

##### *(E)*-*N*-*(4*-*Methoxybenzylidene)*-*1H*-*benzo[d]imidazol*-*2*-*amine* (**9**)

IR (KBr cm^−1^): 3340 (N–H str., of imidazole ring), 2970 (C–H aromatic ring str.), 1496 (Ar., C=C str.), 1566 (N=CH str.), 1257 (C–O–C str.); ^1^H NMR (DMSO): 9.383 (s, 1H, N=CH), 7.027–7.960 (m, 8H, ArH), 3.523 (s, 3H, OCH_3_), 12.497 (s, 1H, NH of imidazole); ^13^C NMR (DMSO): 57, 114, 115, 123, 126, 130, 138, 162; MS: *m/z* = 252.28 (M^+^ +1).

##### *(E)*-*5*-*((1H*-*Benzo[d]imidazol*-*2*-*yl)imino)pentanal* (**10**)

IR (KBr cm^−1^): 3426 (N–H str.), 3054 (Ar., C-H str.), 1562 (C=C str.), 1623 (N=CH str.), 2773 (Aliphatic C–H str.), 1724 (Aliphatic aldehyde C=O str.); ^1^H NMR (DMSO): 8.454 (t, 1H, N=CH), 6.856–7.143 (m, 4H, ArH), 1.243–2.567 (m, 6H, CH_2_), 9.700 (t, 1H, CH = O); ^13^C NMR (DMSO): 18, 28, 44, 115, 123, 138, 160, 162, 202. MS: *m/z* = 216.26 (M^+^ +1).

##### *(E)*-*N*-*(3,4,5*-*Trimethoxybenzylidene)*-*1H*-*benzo[d]imidazol*-*2*-*amine* (**11**)

IR: 3429 (N–H str.), 3064 (Ar., C–H str.), 1577 (C=C str.), 1606 (N=CH str.), 2835 (Ar., O–CH_3_ str.); ^1^H NMR (DMSO): 9.476 (s, 1H, N=CH), 6.962–7.859 (m, 6H, ArH), 10.462 (s, 1H, NH of imidazole) 3.382 (s, 9H, (OCH_3_)_3_); ^13^C NMR (DMSO): 56, 106, 115, 123, 127, 138, 141, 152, 159, 162; MS: *m/z* = 312.14 (M^+^ +1).

##### *(E)*-*1*-*(2*-*(Ethylideneamino)*-*1H*-*benzo[d]imidazol*-*1*-*yl)ethanone* (**12**)

IR (KBr cm^−1^): 1661 (C=O str.), 2919 (C–H aromatic str.), 1575 (N=CH str.), 2849 (CH str. (sym), R-CH_3_); ^1^H NMR (DMSO): 7.305–7.627 (m, 4H, Ar–H), 7.233 (s, 1H, N=CH), 1.273–1.276 (d, 3H, CH_3_), 2.856 (s, 3H, CH_3_); ^13^C NMR (DMSO): 16, 24, 115, 123, 129, 138, 141, 162, 168; MS: *m/z* = 201 (M^+^ +1).

##### *(E)*-*(3,5*-*Dinitrophenyl)(2*-*((4*-*methoxy*-*benzylidene)amino)*-*1H*-*benzo[d]imidazol*-*1*-*yl)methanone* (**13**)

IR (KBr cm^−1^): 1710 (C=O str.), 2924 (C–H aromatic str.), 1537 (N=CH str.), 1545 (Ar-NO_2_ str.), 1110 (C–O–C str., OCH_3_); ^1^H NMR (DMSO): 6.785–7.943 (m, 8H, ArH), 8.632 (s, 1H, N=CH), 2.984 (s, 3H, (OCH_3_), 8.912–9.063 (m, 3H, Ar(NO_2_)_2_); ^13^C NMR (DMSO): 56, 115, 123, 125, 130, 150, 163, 168; MS: *m/z* = 445 (M^+^ +1).

##### *(E)*-*5*-*((1*-*(3,5*-*Dinitrobenzoyl)*-*1H*-*benzo[d]imidazol*-*2*-*yl)imino)pentanal* (**14**)

IR (KBr cm^−1^): 1701 (C=O str.), 3122 (C–H aromatic str.), 1627 (N=CH str.), 1543 (Ar., NO_2_ str.), 1727 (Aliphatic aldehyde C=O str.); ^1^H NMR (DMSO): 6.875-7.946 (m, 4H, ArH), 8.632 (s, 1H, N=CH), 8.912–9.063 (m, 3H, Ar(NO_2_)_2_), 9.254–9.678 (m, 1H, CHO); ^13^C NMR (DMSO): 19, 28, 44, 115, 123, 125, 130, 150, 163, 168; MS: *m/z* = 409 (M^+^ +1).

##### *(E)*-*5*-*((1*-*Acetyl*-*1H*-*benzo[d]imidazol*-*2*-*yl)imino)pentanal* (**15**)

IR (KBr cm^−1^): 1695 (C=O str.), 3050 (C–H aromatic str.), 1606 (N=CH str.), 1535 (C-NO_2_ str.), 2860 (C–H sym. str., R-CH_3_), 1728 (Aliphatic aldehyde C=O str.); ^1^H NMR (DMSO): 7.875–8.246 (m, 4H, ArH), 7.632 (s, 1H, N=CH), 9.254–9.678 (m, 1H, CHO), 2.856 (s, 3H, CH_3_); ^13^C NMR (DMSO): 19, 24, 28, 44, 115, 123, 138, 142, 163, 168, 202; MS: *m/z* = 257 (M^+^ +1).

##### *(E)*-*(3,5*-*Dinitrophenyl)(2*-*((4*-*hydroxybenzylidene)amino)*-*1H*-*benzo[d]imidazol*-*1*-*yl)methanone* (**16**)

IR (KBr cm^−1^): 1685 (C=O str.), 3094 (C–H aromatic str.), 1630 (N=CH str.), 1544 (C–NO_2_ str.), 3465 (O–H str); ^1^H NMR (DMSO): 6.885–7.632 (m, 8H, ArH), 8.654 (s, 1H, N=CH), 8.912–9.063 (m, 3H, Ar (NO_2_)_2_); ^13^C NMR (DMSO): 115, 123, 125, 130, 132, 138, 150, 160, 168; MS: *m/z* = 431 (M^+^ +1).

##### *(E)*-*(2*-*((2,4*-*Dimethoxybenzylidene)amino)*-*1H*-*benzo[d]imidazol*-*1*-*yl)(naphthalen*-*2*-*yl)methanone* (**17**)

IR (KBr cm^−1^): 1695 (C=O str.), 2919 (C–H aromatic str.), 1634 (N=CH str.), 2850 (Ar., OCH_3_ str.); 1646 (naph. ring str.); ^1^H NMR (DMSO): 6.844–8.213 (m, 14H, ArH), 8.612 (s, 1H, N=CH), 2.804 (s, 6H, (OCH_3_)_2_); ^13^C NMR (DMSO): 56, 101, 107, 109, 115, 123, 127, 129, 132, 138, 142, 160, 168; MS: *m/z* = 435 (M^+^ +1).

##### *(E)*-*Naphthalen*-*2*-*yl(2*-*((3,4,5*-*trimethoxybenzylidene)amino)*-*1H*-*benzo[d]imidazol*-*1*-*yl)methanone* (**18**)

IR (KBr cm^−1^): 1691 (C=O str.), 1548 (N=CH str.), 1140 (C–O–C str., OCH_3_); 795 (C–H out of plane bending, naphthalene ring); ^1^H NMR (DMSO): 8.590 (s, 1H, N=CH), 4.194 (s, 9H, (OCH_3_)_3_), 6.971–8.070 (m,11H, Ar–H); ^13^C NMR (DMSO): 57, 107, 115, 123, 124, 127, 128, 131, 139, 142, 151, 160, 168; MS: *m/z* = 465 (M^+^ +1).

##### *(E)*-*(3,5*-*Dinitrophenyl)(2*-*((3,4,5*-*trimethoxybenzylidene)amino)*-*1H*-*benzo[d]imidazol*-*1*-*yl)methanone* (**19**)

IR (KBr cm^−1^): 1681(C=O str.), 1539 (N=CH str.), 2850 (CH_3_ sym. str., R-OCH_3_); 1345 (C–NO_2_ str.); ^1^H NMR (DMSO): 8.947 (s, 1H, N=CH), 3.955 (s, 9H, (OCH_3_)_3_), 9.860–9.865 (m, 3H, Ar-(NO)_2_), 7.948–7.951 (d, 2H, Ar–H), 7.343–7.366 (m, 2H, Ar–H); ^13^C NMR (DMSO): 57, 106, 115, 125, 128, 129, 131, 139, 142, 147, 151, 168; MS: *m/z *= 505 (M^+^ +1).

##### *(E)*-*1*-*(2*-*((3,4,5*-*trimethoxybenzylidene)amino)*-*1H*-*benzo[d]imidazol*-*1*-*yl)hexadecan*-*1*-*one* (**20**)

IR (KBr cm^−1^): 1685 (C=O str.), 3061 (C–H aromatic str.), 1623 (N=CH str.), 2843 (Ar., O–CH_3_ str.), 1266 (Palmitoyl group str.); ^1^H NMR (DMSO): 7.283–7.286 (m, 6H, ArH), 1.278–2.386 (m, 28H, CH_2_ of palmitoyl), 0.884–0.903 (t, 3H, CH_3_), 3.264 (s, 9H, (OCH_3_)_3_); ^13^C NMR (DMSO): 14, 23, 26, 30, 32, 56, 106, 115, 123, 128, 130, 139, 142, 160, 170; MS: *m/z* = 549 (M^+^ +1).

##### *(E)*-*(3*-*Nitrophenyl)(2*-*((4*-*(pyridin*-*2*-*yl)benzylidene)amino)*-*1H*-*benzo[d]imidazol*-*1*-*yl) methanone* (**21**)

IR (KBr cm^−1^): 1682 (C=O str.), 2922 (C–H aromatic str.), 1525 (N=CH str.), 1557 (C=C and C=N str. of pyridine ring), 1543 (C–NO_2_ str.); ^1^H NMR (DMSO): 10.019 (s, 1H, N=CH), 7.305–8.658 (m, 15H, ArH), 8.662 (s, 1H, Ar–NO_2_); ^13^C NMR (DMSO): 115, 123, 126, 129, 130, 132, 142, 150, 155, 160, 168; MS: *m/z* = 447 (M^+^ +1).

##### *(2*-*((E)*-*((E)*-*3*-*(4*-*(Dimethylamino)phenyl)allylidene)amino)*-*1H*-*benzo[d]imidazol*-*1*-*yl)(3*-*nitrophenyl)methanone* (**22**)

IR (KBr cm^−1^): 1723(C=O str.), 2920 (C–H aromatic str.), 1530 (N=CH str.), 1549 (Ar-NO_2_ str.), 1349 (C–N str., of ter. arylamine); ^1^H NMR (DMSO): 8.390–8.410 (d, 1H, N=CH), 6.731–6.740 (d, 1H, –CH = CH), 6.250–8.355 (m, 11H, ArH), 8.919 (s, 1H, Ar-NO_2_) 3.559 (s, 6H, (CH_3_)_2_); ^13^C NMR (DMSO): 40, 115, 123, 127, 131, 138, 149, 164, 168; MS: *m/z* = 439 (M^+^ +1).

##### *(E)*-*(2*-*((4*-*(Dimethylamino)benzylidene)amino)*-*1H*-*benzo[d]imidazol*-*1*-*yl)(3*-*nitrophenyl) methanone* (**23**)

IR (KBr cm^−1^): 1719 (C=O str.), 3085 (C–H aromatic str.), 1615 (N=CH str.), 1545 (Ar-NO_2_ str.), 1514 (C–N str.); ^1^H NMR (DMSO): 7.169–8.987 (m, 12H, ArH), 9.568 (s, 1H, N=CH), 2.909 (s, 6H (CH_3_)_2_); ^13^C NMR (DMSO): 40, 115, 123, 127, 131, 138, 149, 160, 168; MS: *m/z* = 413 (M^+^ +1).

##### *(E)*-*(2*-*(((2*-*hydroxynaphthalen*-*1*-*yl)*-*methylene)amino)*-*1H*-*benzo[d]imidazol*-*1*-*yl)(3*-*nitrophenyl)methanone* (**24**)

IR (KBr cm^−1^): 1696 (C=O str.), 2924 (C–H aromatic str.), 1553 (N=CH str.), 1546 (Ar-NO_2_ str.), 752 (O–H bending (out of plane)); ^1^H NMR (DMSO): 6.748–8.632 (m, 14H, ArH), 9.652 (s, 1H, N=CH); ^13^C NMR (DMSO):115, 118, 123, 125, 127, 128, 131, 138, 149, 160, 168; MS: *m/z* = 436 (M^+^ +1).

##### *(E)*-*(2*-*((4*-*Nitrobenzylidene)amino)*-*1H*-*benzo[d]imidazol*-*1*-*yl)(3*-*nitrophenyl)methanone* (**25**)

IR (KBr cm^−1^): 1704 (C=O str.), 3107 (C–H aromatic str.), 1617 (N=CH str.), 1549 (Ar., NO_2_ str.); ^1^H NMR (DMSO): 7.206–8.689 (m, 12H, ArH), 9.672 (s, 1H, N=CH); ^13^C NMR (DMSO): 115, 121, 123, 125, 127, 131, 136, 139, 151, 160, 168; MS: *m/z* = 415 (M^+^ +1).

##### *(E)*-*Naphthalen*-*2*-*yl(2*-*((4*-*nitrobenzylidene)amino)*-*1H*-*benzo[d]imidazol*-*1*-*yl)methanone* (**26**)

IR (KBr cm^−1^): 1686 (C=O str.), 3056 (C–H aromatic str.), 1600 (N=CH str.), 1545 (Ar., NO_2_ str.), 1592 (Naphthalene ring str.); ^1^H NMR (DMSO): 6.865–7.954 (m, 11H, ArH), 8.765 (s, 1H, N=CH), 8.923–8.967 (m, 4H, Ar(NO_2_); ^13^C NMR (DMSO): 115, 123, 124, 128, 129, 131, 135, 136, 139, 142, 149, 160, 168; MS: *m/z* = 420 (M^+^ +1).

##### *(E)*-*(2*-*((3,4*-*Dimethoxybenzylidene)amino)*-*1H*-*benzo[d]imidazol*-*1*-*yl)(3*-*nitro phenyl)methanone* (**27**)

IR (KBr cm^−1^): 1684 (C=O str.), 1611 (N=CH str.), 2875 (Ar., O–CH_3_ str.); 1543 (Ar., NO_2_ str.); ^1^H NMR (DMSO): 7.463–8.932 (m, 11H, ArH), 8.185 (s, 1H, N=CH), 2.904 (s, 6H, (OCH_3_)_2_); ^13^C NMR (DMSO): 56, 115, 123, 125, 127, 136, 139, 142, 147, 150, 152, 160, 168; MS: *m/z* = 430 (M^+^ +1).

##### *(E)*-*1*-*(2*-*((4*-*Nitrobenzylidene)amino)*-*1H*-*benzo[d]imidazol*-*1*-*yl)hexadecan*-*1*-*one* (**28**)

IR (KBr cm^−1^): 1685 (C=O str.), 2954 (C–H aromatic str.), 1618 (N=CH str.), 1271 (Palmitoyl group. str.), 1547 (Ar-NO_2_str.); ^1^H NMR (DMSO): 7.624–8.163 (m, 8H, ArH), 8.672 (s, 1H, N=CH), 1.243–2.496 (m, 28H, CH_2_ of palmitoyl), 0.845–0.878 (t, 3H, CH_3_); ^13^C NMR (DMSO): 14, 23, 26, 30, 32, 56, 106, 115, 120, 123, 125, 128, 131, 135, 136, 139, 142, 149, 160, 170; MS: *m/z* = 504 (M^+^ +1).

##### *(E)*-*1*-*(2*-*((4*-*(Dimethylamino)benzylidene)amino)*-*1H*-*benzo[d]imidazol*-*1*-*yl)hexadecan*-*1*-*one* (**29**)

IR (KBr cm^−1^): 2927 (C–H, aromatic str.), 2813 (C–H str. aliphatic), 1659 (C=O str.), 1594 (N=CH str.), 1303 (C–N str.), 1278 (palmitoyl group str.); ^1^H NMR (DMSO): 7.878–7.901 (d, 1H, N=CH), 6.606–6.622 (d, 1H, –CH = CH), 6.661–7.519 (m, 8H, ArH), 3.773 (s, 6H, (CH_3_)_2_), 1.252–2.368 (m, 28H, CH_2_ of palmitoyl), 0.861–0.894 (t, 3H, CH_3_); ^13^C NMR (DMSO): 14, 23, 26, 30, 32, 56, 106, 115, 120, 123, 125, 128, 130, 139, 142, 149, 164, 170; MS: *m/z* = 528 (M^+^ +1).

##### *(E)*-*(2*-*((4*-*(Dimethylamino)benzylidene)amino)*-*1H*-*benzo[d]imidazol*-*1*-*yl)(naphthalen*-*2*-*yl)methanone* (**30**)

IR (KBr cm^−1^): 1683 (C=O str.), 3054 (C–H aromatic str.), 1608 (N=CH str.), 1521 (Ar. NO_2_ str.), 1448 (C–N str.), 778 (C–H out of plane bending, naphthalene ring); ^1^H NMR (DMSO): 6.668–7.985 (m, 15H, ArH), 9.584 (s, 1H, N=CH), 2.909 (s, 6H (CH_3_)_2_); ^13^C NMR (DMSO): 40, 115, 123, 124, 128, 130, 139, 142, 160, 168; MS: *m/z* = 418 (M^+^ +1).

## Biological evaluation

### In vitro antimicrobial assay

Tube dilution method [15] was used to determine the antimicrobial activity of synthesized compounds against Gram-positive bacteria: *Staphylococcus aureus* (MTCC-3160); *Bacillus subtilis* (MTCC-441), the Gram-negative bacterium *Escherichia coli* (MTCC-443) and fungal species: *Candida albicans* (MTCC-227) and *Aspergillus niger* (MTCC-281). Dilutions were made for test and standard compounds in appropriate double strength nutrient broth—I.P. (bacteria) or Sabouraud dextrose broth—I.P. (fungi) [16]. The test and standard compounds were incubated at 37 °C for 24 h (bacteria), at 25 °C for 7 days (*A. niger*) and at 37 °C for 48 h (*C. albicans*) and the minimum inhibitory concentration (MIC) was recorded in µg/mL.

### In vitro anticancer assay

The in vitro anticancer activity of the developed compounds was performed by the Sulforhodamine B (SRB) assay as described by Skehan et al. [14]. The optimal MCF-7 cell count was seeded on flat-bottom well plates and allowed to attach overnight. The compounds (20 μL) were added in quadruplicates and incubated for 72 h (both drug-free control and treated cells). Cells in each well were fixed with 200 μL of 10% cold trichloroacetic acid. After incubation for 30 min, the individual wells were rinsed with water, allowed to stain in 100 µL 0.4% SRB [Sigma-Aldrich, St Louis, Missouri, USA] (*w/v*; in 1% acetic acid) for 15 min. The air-dried plates were placed on a plate shaker and bound SRB was solubilised in 100 µL 10 mM Tris base solution. Absorbance was measured using a spectrophotometer at 570 nm and a dose–response curve was plotted from which the IC_50_ value of each compound against each cell type was determined.

## Conclusion

In conclusion, a series of 1,2-disubstituted benzimidazole derivatives were synthesized and assessed for in vitro antimicrobial and anticancer activities against five representative microbial species and cancer cell line. Antimicrobial activity results indicated that the synthesized compound **1** has promising activity towards Gram negative bacteria *E. coli*. None of the compound showed more potent activity against Gram positive bacteria *B. subtilis* and *S. aureus* when compared to reference drug norfloxacin. Moreover, compounds **2** and **19** showed interesting results against fungal strains *C. albicans* and *A. niger* and comparable to fluconazole. The results from anticancer activity indicated that compounds **12**, **21**, **22** and **29** showed promising activity against MCF7. These active compounds may be taken as lead compounds for discovery of novel antimicrobial and anticancer agents in future.
